# Latex-fruit syndrome and degree of severity of natural rubber latex allergy: is there a link?

**DOI:** 10.1186/2045-7022-1-S1-O18

**Published:** 2011-08-12

**Authors:** Elisabetta Calamelli, Valentina Piccinno, Arianna Giannetti, Giampaolo Ricci, Andrea Pession

**Affiliations:** 1 University of Bologna, Dipartimento di Pediatria, Bologna, Italy

## Background

Latex-fruit syndrome (LFS), defined as hypersensitivity to particular fresh fruits (*Wagner S. & Breiteneder H. Bioch Soc Trans, 2002*), has been described in 30-50% of pts affected from natural rubber latex (NRL) allergy and it is due to IgE Abs that cross-react with similar epitopes on phylogenetically related proteins. Objective: To estimate the prevalence of LFS in a group of NRL allergic children and adolescents and to evaluate a possible correlation with the severity degree of NRL allergy.

## Methods

This retrospective study analyzed 22 pts (17 M, 5 F; mean age 15,2 yrs) referred to the Pediatric Allergology of University of Bologna from Jan. 1990 to Sept. 2010 with a history of IgE-mediated NRL allergy associated with a level of latex specific IgE (sIgE) ≥ 0.35 kU/L (ImmunoCAP 1000, Phadia; Sweden) and/or a positive response to Skin Prick Test (wheal ≥ 3 mm) with latex extract (Lofarma, Milan; Italy). Levels of total and sIgE to grass pollen and to the main fruits implicated in LFS were analysed.

## Results

The mean age at diagnosis of NRL allergy was 7,2 yrs (range 3-12). NRL allergic pts were divided in 2 groups, according to the severity of symptoms after latex contact. In group A were included 13 pts (59%) with mild symptoms (contact urticaria); in group B the remaining 9 subjects (41%) with moderate-severe symptoms (generalized urticaria w/wo angioedema and/or respiratory symptoms and/or anaphylaxis). Eight of the 22 subjects (36,4%) reported symptoms from LFS to the following fruits or combination of its: kiwi (5), peach (2), chestnut (2), cherry (1), apple (1), melon (1). The prevalence of LFS was higher in group B than in group A (respectively 7/9, 78% vs. 1/13, 8%; p<.005, Chi-square test). No statistically differences in median values of total and sIgE were found between the 2 groups (Mann-Whitney U test).

## Conclusions

Our study confirms a relevant prevalence of LFS (36,4%) in patients with NLR, with kiwi, alone or in combination, as the main implicated fruit. Moreover, a significantly higher prevalence of LFS in subjects with more severe manifestations of NRL allergy was documented.

